# Supplementary material for: phylobar: an R package for multiresolution compositional barplots in omics studies
Source: Bioinformatics. 2026 Mar 25;42(4):btag151. doi: 10.1093/bioinformatics/btag151 (PMC13105843; doi:10.1093/bioinformatics/btag151)
Supplement: btag151_Supplementary_Data [file btag151_supplementary_data.pdf]

Supporting Information for "phylobar: an R package for multiresolution compositional barplots in omics studies"

Megan Kuo[1], Kim-Anh Lê Cao[2], Saritha Kodikara[2,*], Jiadong Mao[2,*], and Kris Sankaran[1,*]

[1]Department of Statistics, University of Wisconsin–Madison, USA
[2]Melbourne Integrative Genomics, School of Mathematics and Statistics, University of Melbourne, Australia
[*]*Co-corresponding authors.*

1 Comparison with Other Software

Tool	Interactive	Multiscale	Preserves phylogeny	Shows sample variation	Compare levels at once
phylobar	Yes	Yes	Yes	Yes	Yes
StackbarExtend	No	Partly ($\leq$ 2 levels)	No	Yes	No
krona	Yes	Yes	Yes	No	Yes
microbiomeExplorer	Yes	Yes	Yes	Yes	No

Table 1: Comparison of visualization tools for microbiome compositional data.

Several software packages support multiscale composition visualization in microbiome data. We compare these alternatives with phylobar (Table 1). StackbarExtend [Cuisiniere and Santos, 2024] creates a static ggplot2-based stacked barplot that encodes two taxonomic scales simultaneously (Supplementary Figure 2a). This is done with a hierarchical color palette: coarse-level taxa map to distinct hues (e.g., "red") while finer nested taxa map to shades within those hues. Broad color swaths reveal coarse-level patterns and shade differences within each hue distinguish finer-level taxonomic variation. This approach has the advantage of presenting meaningful patterns without user interaction, and outputs are easily customizable through ggplot2 themes. However, it is limited to two taxonomic levels, and when finer levels contain many categories, shades become difficult to distinguish. StackbarExtend may therefore be effective in simpler cases but lacks phylobar's flexibility in more complex data or queries.

Krona [Ondov et al., 2011] gives an interactive sunburst for taxonomic composition. Each radial layer represents one taxonomic level and segment widths encode read proportion. As in StackbarExtend, nested taxa are given different shades of the same hue. Hovering highlights a segment and its descendants. Single-clicking shows a group's proportion within its parent. Double-clicking filters to that group's descendants and rescales segments to fill the sunburst

area. The main advantage is that double-clicking updates the view to dedicate all visual space to the category of interest. However, this necessarily discards context from other taxa. Further, Krona displays only a single sample (or an average across samples), precluding the sample-to-sample comparisons possible with stacked barplots. Like StackbarExtend, color shades become difficult to distinguish when many taxa are shown at once.

microbiomeExplorer [Reeder et al., 2020] provides an interactive stacked barplot as a part of a broader exploratory data analysis toolkit. A dropdown menu allows viewers to select a taxonomic level, updating the barplot categories accordingly. Rare taxa are collapsed into an "Other" category to keep the color palette manageable. This approach supports sample-to-sample comparisons and allows interactive comparison of abundances across taxonomic scales, with the extra benefit of integration into a larger microbiome analysis pipeline. However, collapsing rare taxa into "Other" can obscure low-abundance groups, and multiscale comparison places demands on the viewer's memory – abundances at one level are lost from view when switching between levels.

We additionally note that phylobar applies to either taxonomic hierarchies or phylogenetic trees, while the alternatives discussed above are tailored to taxonomic hierarchies.

2 Runtime Evaluation

Supplementary Figure 3 presents runtime of the `phylobar` function on simulated data with varying sample sizes and feature dimensions. Data are generated with random Poisson counts and the tree is generated with the `rtree()` function in the ape package Paradis et al. [2004]. By default, the hierarchical clustering used to sort samples in the stacked barplot dominates runtime when the sample size is large (right panel). Disabling this option – sorting samples by their input order instead – greatly reduces runtime (left panel).

Even when the `phylobar` function executes quickly, the interpretability and rendering performance can degrade. At large sample sizes, bars become too narrow to distinguish, and SVG rendering lags as the number of drawn rectangles grows (there is a rectangle for every sample × taxonomic group visible in the tree). In this setting, we recommend using `subset_cluster` to select representative samples or else splitting samples into groups and creating separate a phylobar plot per group. With this approach, even 1000 taxa can be visualized with smooth browser interaction, as documented in the article "Comparing Runtime across Sample Sizes and Number of Taxa" on the documentation homepage.

References

Thibault Cuisiniere and Manuela Santos. Stackbarextended: a user-friendly stacked bar-plot representation incorporating phylogenetic information and microbiota differential abundance analysis. *F1000Research*, 13:914, August 2024. ISSN 2046-1402. doi: 10.12688/f1000research.151662.1. URL `http://doi.org/10.12688/f1000research.151662.1`.

Brian D Ondov, Nicholas H Bergman, and Adam M Phillippy. Interactive metagenomic visu-

alization in a web browser. *BMC Bioinformatics*, 12(1), September 2011. ISSN 1471-2105. doi: 10.1186/1471-2105-12-385. URL `http://doi.org/10.1186/1471-2105-12-385`.

Emmanuel Paradis, Julien Claude, and Korbinian Strimmer. Ape: Analyses of phylogenetics and evolution in r language. *Bioinformatics*, 20(2):289–290, January 2004. ISSN 1367-4803. doi: 10.1093/bioinformatics/btg412. URL `http://doi.org/10.1093/bioinformatics/btg412`.

Janina Reeder, Mo Huang, Joshua S Kaminker, and Joseph N Paulson. MicrobiomeExplorer: an r package for the analysis and visualization of microbial communities. *Bioinformatics*, 37(9):1317–1318, October 2020. ISSN 1367-4811. doi: 10.1093/bioinformatics/btaa838. URL `http://doi.org/10.1093/bioinformatics/btaa838`.

a. StackbarExtend

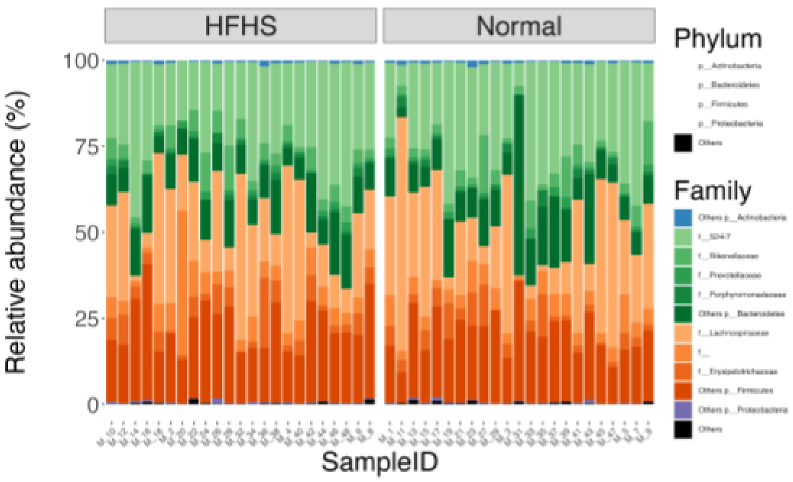

b. krona

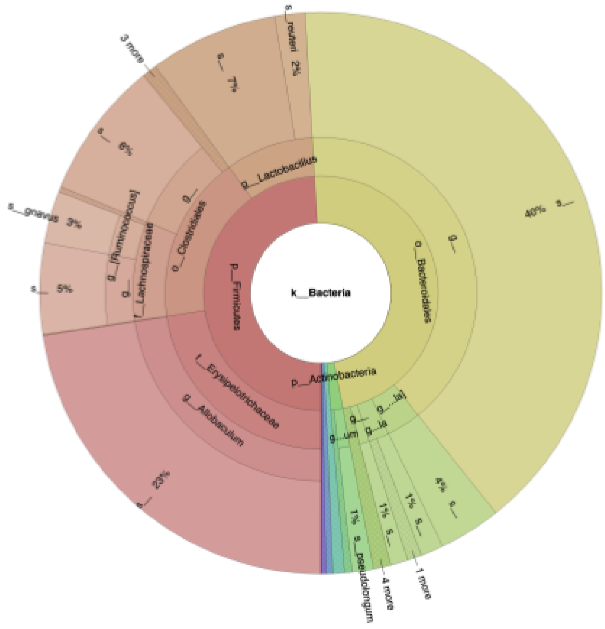

Figure 1: Existing packages applied to the HFHS data from Figure 1a-c of the main manuscript. (a) StackbarExtend allows static comparison of two taxonomic levels, in this case Order and Family. The coarser level is shown as a broad color choice and the finer level is variation in shade within that color. (b) The krona package creates interactive pie charts for individual samples. Clicking on one segment reveals further finer-level taxonomic variation within that segment.

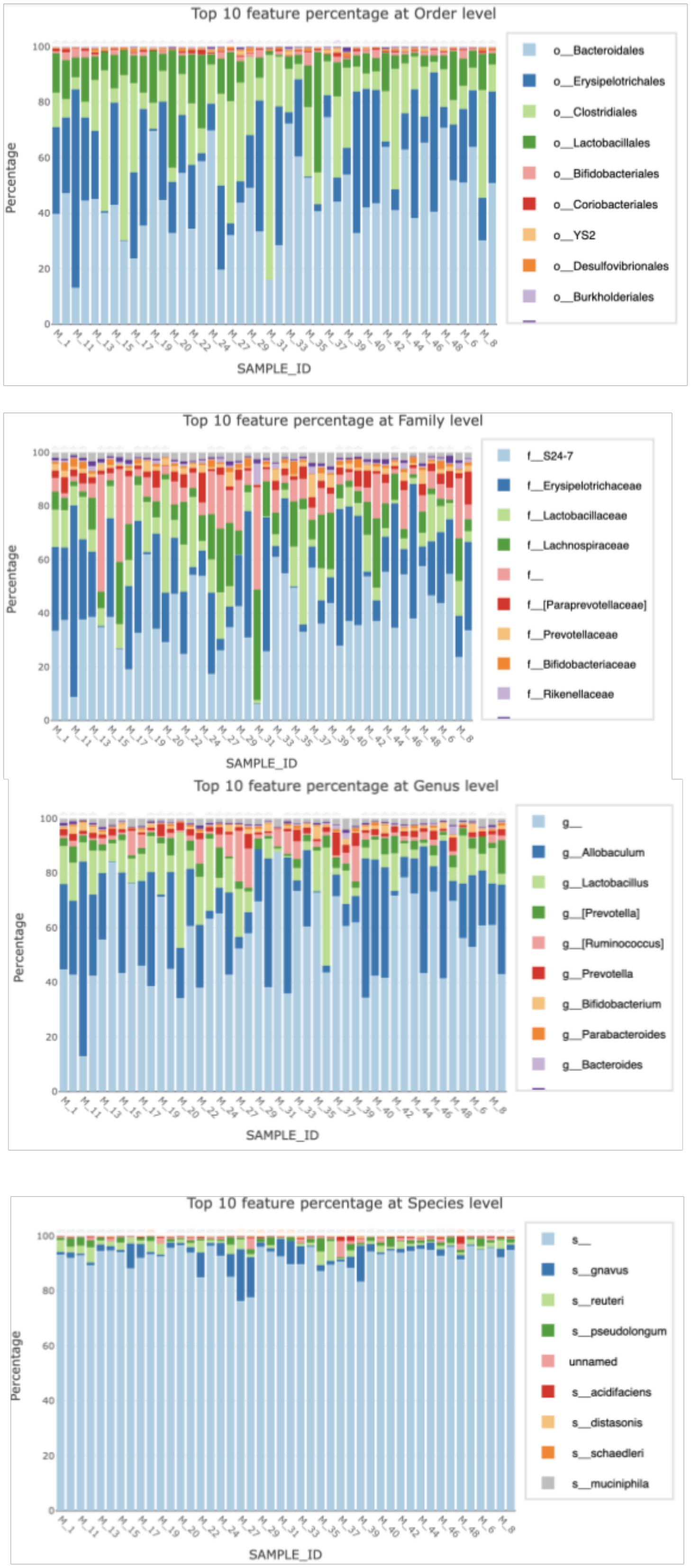

Figure 2: A screenshot from the interactive microbiomeExplorer package. A dropdown menu (not shown) can be used to change the taxonomic level in the stacked bar plot, resulting in the four variations given here.

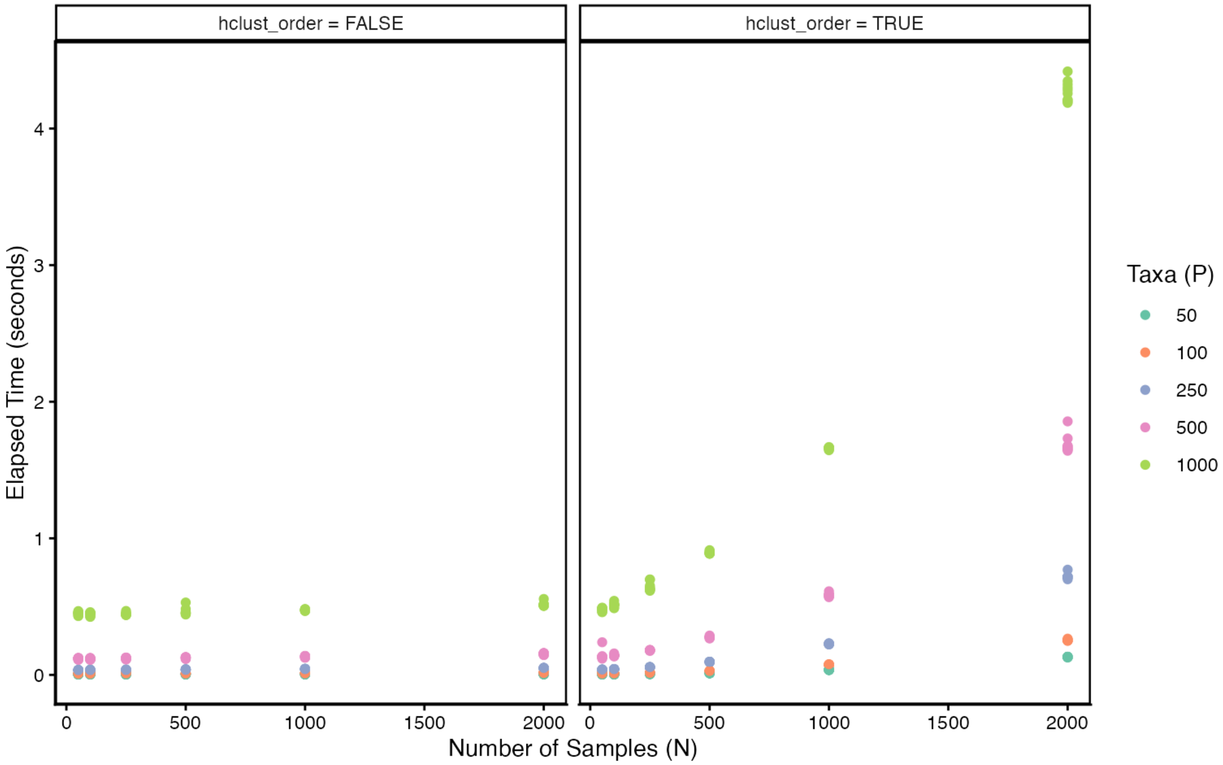

Figure 3: The runtime of the `phylobar` function across varying sample sizes and number of taxonomic features. By default phylobar orders samples in the stacked barplot using hierarchical clustering, which dominates runtime when the number of taxa is large.
